# One-Step Fabrication of Hollow Spherical Cellulose Beads: Application in pH-Responsive Therapeutic Delivery

**DOI:** 10.1021/acsami.1c19577

**Published:** 2022-01-11

**Authors:** Tamilselvan Mohan, Urban Ajdnik, Chandran Nagaraj, Florian Lackner, Andreja Dobaj Štiglic, Thirvengadam Palani, Lunjakorn Amornkitbamrung, Lidija Gradišnik, Uroš Maver, Rupert Kargl, Karin Stana Kleinschek

**Affiliations:** † Institute for Chemistry and Technology of Biobased Systems (IBioSys), Graz University of Technology, Stremayrgasse 9, 8010 Graz, Austria; ‡ Faculty of Mechanical Engineering, Institute of Engineering Materials and Design, University of Maribor, Smetanova 17, 2000 Maribor, Slovenia; § Ludwig Boltzmann Institute for Lung Vascular Research, Stiftingtalstrasse 24, 8010 Graz, Austria; ∥ School of Chemistry and Chemical Engineering and State Key Laboratory of Metal Matrix Composites, Shanghai Jiao Tong University, 800 Dongchuan Road, Shanghai 200240, China; ⊥ Faculty of Engineering, Department of Chemical Engineering Research Unit in Polymeric Materials for Medical Practice Devices, Chulalongkorn University, 254 Phayathai Rd, Bangkok 10330, Thailand; # Faculty of Medicine, Department of Pharmacology, University of Maribor, Taborska ulica 8, 2000 Maribor, Slovenia

**Keywords:** cellulose acetate, beads, deacetylation, hollow structure, drug delivery, targeted release, diclofenac, fibroblast cells

## Abstract

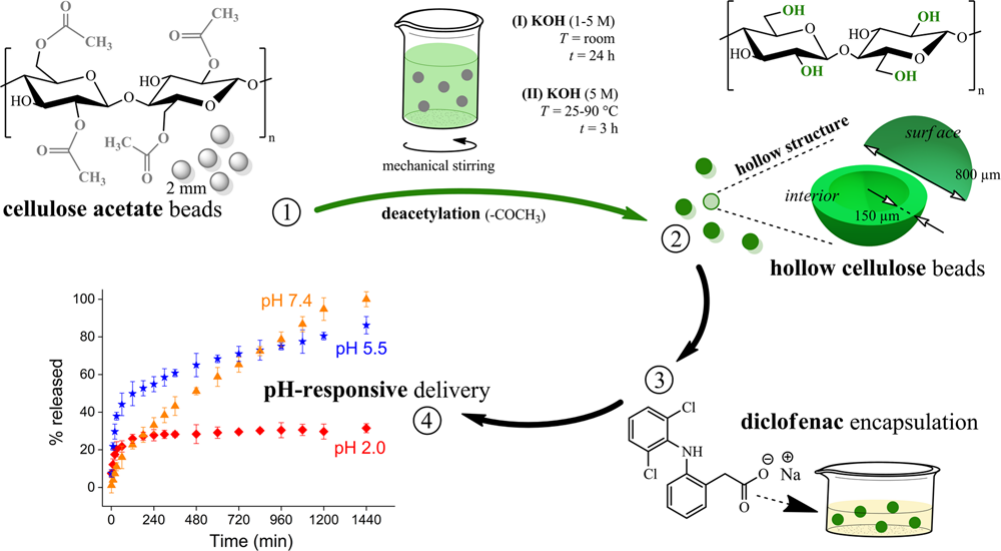

The path to greater sustainability and the development of polymeric drug delivery systems requires innovative approaches. The adaptation and use of biobased materials for applications such as targeted therapeutic delivery is, therefore, in high demand. A crucial part of this relates to the development of porous and hollow structures that are biocompatible, pH-responsive, deliver active substances, and contribute to pain relief, wound healing, tissue regeneration, and so forth. In this study, we developed a facile single-step and water-based method for the fabrication of hollow spherical cellulose beads for targeted drug release in response to external pH stimuli. Through base-catalyzed deprotection, hydrophobic solid and spherical cellulose acetate beads are transformed into hydrophilic cellulose structures with a hollow interior (wall thickness: 150 μm and inner diameter: 650 μm) by a stepwise increment of temperature and treatment time. Besides the pH-responsive fluid uptake properties, the hollow cellulose structures exhibit a maximum encapsulation efficiency of 20–85% diclofenac (DCF), a nonsteroidal anti-inflammatory drug, used commonly to treat pain and inflammatory diseases. The maximum amount of DCF released *in vitro* increased from 20 to 100% when the pH of the release medium increased from pH 1.2 to 7.4. As for the DCF release patterns and kinetic models at specific pH values, the release showed a diffusion- and swelling-controlled profile, effortlessly fine-tuned by external environmental pH stimuli. Overall, we show that the modified beads exhibit excellent characteristics for transport across the gastrointestinal tract and enhance the bioavailability of the drug. Their therapeutic efficacy and biocompatibility are also evident from the studies on human fibroblast cells. We anticipate that this platform could support and inspire the development of novel sustainable and effective polysaccharide-based delivery systems.

## 1 Introduction

Stimuli-responsive release carriers exhibit altered properties following changes in environmental variables, typically pH, temperature, salt concentration, enzymes, redox (oxidation–reduction), and light.^1−4^ Being sensitive to the physiological environment, these carrier materials enable the design of “smart” and controlled drug release systems. Stimuli triggers can be categorized into organ-level triggers, related to pathophysiological changes and cellular compartment-specific triggers.^1^ Although the potential environmental triggers for medical applications are limited, variations in the pH of different cellular compartments, tissues, and organs offer pH as an accessible stimulus. The change in the external pH acts as a stimulus, to which the response is observed in the form of a change in the properties of the selected responsive material. pH-responsive materials undergo physical and chemical changes, resulting in swelling, shrinkage, dissociation, degradation, or membrane fusion and disruption. This is triggered by either the protonation of ionizable groups or degradation of acid-cleavable bonds in the pH-responsive materials.^1^ For instance, carrier materials bearing −C(=O)OH functional groups demonstrate higher solubility at the basic pH range and can be used to protect low pH-sensitive medications, for example, intestine-targeted delivery.^5,6^ Every oral delivery must consider the change in pH along the gastrointestinal tract, including saliva (6.0–7.0), gastric fluid (1.0–3.5), bile (7.8), pancreatic fluid (8.0–8.3), small intestinal fluid (7.8–8.0), and large intestinal fluid (5.5–7.0).^1,7^ Additionally, the nominal pH range of chronic wounds and inflamed tissues has been reported between 7.4 and 5.4, while cancerous tissues have a more acidic extracellular pH in the microenvironment of a tumor.^8−11^

However, many drugs cannot sustain the gastrointestinal environment.^12^ Hence, targeted drug delivery with simultaneous drug protection is among the prime goals of drug delivery research targeting this organ. Among drug carriers, spherical beads (in mm or μm) with unique properties, such as high porosity, large available specific surface area, low density, and adjustable chemistry, provide an exceptional template for drug delivery.^13^ These spherical beads also comply with durability and are easily transported and stored. Various types of hollow beads have been fabricated from a plethora of materials for diverse applications (*e.g.*, biomedical, environmental, biosensing, insulation, and catalysis), ranging from carbon (*e.g.,* nanotubes and graphene)^14−17^ to inorganic oxides [*e.g.*, titanium dioxide, silicon dioxide, and aluminum(III) oxide]^18−22^ to synthetic polymers (*e.g.,* polyamide and polyurethane)^23−27^ and natural polymeric materials (*e.g.,* cellulose, chitosan, and proteins).^28−32^ Polymers, as one of the most extensively utilized materials for this purpose, have made remarkable advances in drug delivery and offer several advantages (pH-responsive). In addition to the stable physicochemical properties offered by an ideal polymeric delivery carrier, it should be biocompatible, cost-effective, and protect the incorporated drug. Polymers applied in drug delivery primarily reduce degradation, immunogenicity and toxicity (burst release); improve circulation time; and serve as passive targeting agents. pH-responsive polymers used in medicine must also respond to a range of biological conditions by a change in solubility, swelling, conformation, degree of ionization, and release of an active compound.

Polysaccharide-based beads are a new generation of porous polymeric materials that exhibit outstanding properties compared to synthetic-based beads, while being regarded as the most sustainable.^33−36^ These polysaccharide-formulated beads have become attractive as carriers in drug delivery, owing to the abundance of the raw material, relatively inexpensive production, and intrinsic properties: biocompatibility, swelling capacity, biodegradability, and positive environmental factors (sustainability and renewability).^37^ Furthermore, the functional groups carried by polysaccharides [*e.g.*, −C(=O)OH, −OH, and −NH_3_] can be fine-tuned to shape and advance their properties as delivery carriers.^38^ Cellulose beads are high-performance materials used in a large range of applications.^35,36^ Cellulose is not only the most abundant renewable biopolymer^39^ but also a promising and comprehensively investigated natural material, covering applications ranging from water treatment to tissue engineering,^40^ packaging,^41^ biosensors,^42^ and drug delivery.^43^ The high porosity with internal surface area,^44,45^ gradual release,^46,47^ and porosity of the matrix^48^ make cellulose beads an attractive absorbent and competitive granulate material for biomedical applications (*e.g.,* oral drug delivery^28^). Importantly, cellulose enables the drug itself to be stabilized in an amorphous state with high stability toward recrystallization.^49^ Compared to the synthetic polymer-derived beads, cellulose-based beads (size: mm or μm) show higher mechanical stiffness and are relatively more simple to manufacture.^50−52^ In recent decades, various methods for the preparation of cellulose beads have been reported, including (1) dissolution of cellulose, (2) spheronization, and (3) coagulation.^53^ In this study, we show for the first time the fabrication of polysaccharide cellulose beads with a hollow interior, which can be an advantage to achieve high encapsulation and release efficiency for therapeutic drug molecules. Especially, a single-step base-catalyzed deacetylation method to obtain hollow and porous cellulose beads from the commercial cellulose acetate (CA) spherical beads as drug carriers has never been reported. We chose diclofenac (DCF) as the model drug component to demonstrate the encapsulation and pH-responsive release efficiency of the hollow cellulose beads. DCF is a nonsteroid anti-inflammatory drug that is widely used in relieving pain, fighting fever, and decreasing inflammation.^54^ Although modification of cellulose beads has been around for decades,^53^ it commonly adds to the economic and environmental expenditures.^55^

Inspired by all the abovementioned methods, we present a straightforward and scalable method to design cellulose beads and improve their functionality. In this method, the commercially available solid CA spherical beads were transformed into cellulose beads with a porous surface and hollow interior through a simple and environmentally friendly base-catalyzed deprotection method by the fine-tuning of treatment time and temperature. Consequently, due to the increased swelling capacity of cellulose beads in water, increased active substances like DCF can be encapsulated into the beads, and, correspondingly, a sustained and longer-release profile can be achieved. To investigate the applicability, the drug of choice, DCF, was used in a controlled release triggered by the pH of the environment (pH 1.2–7.4). The beads were characterized before and after encapsulation, as well as after *in vitro* release, concerning their morphology, structure, swelling capacity, and release kinetics using different kinetic models. To show the potential of the prepared beads for biomedical applications, their biocompatibility was further tested against human fibroblast cells.

## 2 Experimental Section

### 2.1 Materials

White, spherical CA beads (diameter: 2 mm; density: 1.3 g/cm^3^) were purchased from Cospheric, USA. DCF sodium salt, 5-([4,6-dichlorotriazin-2-yl]amino) fluorescein hydrochloride (DTAF), sodium bicarbonate, potassium hydroxide (KOH) pellets, disodium phosphate heptahydrate (Na_2_HPO_4_·7 H_2_O), and sodium dihydrogen phosphate monohydrate (NaH_2_PO_4_·H_2_O) were purchased from Sigma-Aldrich, Austria, and used as received. Advanced Dulbecco’s modified Eagle’s medium (DMEM) and fetal bovine serum (FBS) were purchased from Thermo Fisher, Germany. Human-derived skin fibroblasts (ATCC CCL-119, Detroit 551, LGC Standard) were purchased from ATCC, UK. Ultrapure water (18.2 MΩ cm) from a Milli-Q water purification system (Millipore Corporation, USA) was used for deacetylation, buffer preparation, and drug release experiments.

### 2.2 Fabrication of Deacetylated Cellulose Acetate Beads

Two different methods were used for the deacetylation of CA beads. Method I: 20 CA beads were immersed in 100 mL of potassium hydroxide solution (KOH: 1 and 5 M) for 24 h at room temperature and stirred continuously (300 rpm) with a mechanical stirrer. Later, they were immersed in 100 mL of water for 2 h, rinsed extensively with water, and dried at room temperature for 12 h. Method II: 20 CA beads were immersed in 100 mL of 5 M KOH for 3 h at different temperatures (25, 50, 80, and 90 °C) and stirred continuously with a mechanical stirrer (300 rpm). Subsequently, the beads were immersed in 100 mL of water for 2 h and then rinsed extensively with water and dried for 12 h at room temperature. The completely deacetylated cellulose acetate (DCA) beads at 90 °C (as confirmed by infrared spectroscopy; see Section 3.1.1) were used for swelling, drug encapsulation, and release studies.

### 2.3 Attenuated Total Reflection Fourier Transform Infrared Spectroscopy

The chemical structure/composition of the CA and deacetylated beads (before and after drug encapsulation) were analyzed using a Bruker Alpha attenuated total reflection Fourier transform infrared (ATR–FTIR) spectrometer at a scan range of 4000–650 cm^–1^. A total of 32 scans were performed with a resolution of 4 cm^–1^. As shown in eqs 1 and 2, we estimated the degree of substitution (DS) (or acetylation) of CA and deacetylated beads by the method described by El Nemr *et al.*(56)
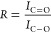
1
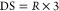
2where *R* is the ratio between the intensities of vibrations; *I*_C=O_ represents the intensity of the ester functional group at 1730 cm^–1^; *I*_C–O_ represents the intensity of C–O stretching in the cellulose backbone at 1030 cm^–1^; and DS is the degree of substitution (or acetylation).

### 2.4 Scanning Electron Microscopy

The microscopic morphology of all bead samples was analyzed using a Carl Zeiss FE-SEM SUPRA 35 VP scanning electron microscope. The scanning electron microscopy (SEM) images were recorded with an acceleration voltage of 1 kV. The samples were mounted on aluminum sample holders, and no sputtering was performed on the samples’ surfaces.

### 2.5 Confocal Laser Scanning Microscopy

The CA and deacetylated beads (surface and interior) were stained with 100 mL of DTAF solution (*c* = 0.1 mg/mL, dissolved in 100 mM bicarbonate buffer, pH 9). 10 CA and DCA beads were left to react for 30 min in a dark place under the exclusion of light. Afterward, the beads were immersed in water for 60 min and then rinsed extensively with water, dried, and stored at room temperature under the exclusion of light. A confocal laser scanning microscope (Leica TCS SP5 II laser scanning confocal microscope equipped with LAS AF imaging software, Leica Microsystems, Germany) was used to observe the surface morphology of the stained samples. The DTAF dye was excited at 495 nm, and the emission was recorded at 516 nm. The image size was 512 × 512 pixels, and the images were scanned at a scan speed of 290 frames s^–1^.

### 2.6 Swelling Studies

Swelling studies were performed for the DCA beads (prepared *via* method II at 90 °C) in three different aqueous media: simulated gastric fluid [SGF, pH 1.2, mixed with 0.2 M HCl and 0.2 M KCl, 1.7:1.0 (v/v)], phosphate-buffered saline (PBS) with pH 5.5, and PBS with pH 7.4. Accurately weighed amounts of beads (2.5 g) were put in a sphere lattice (sieve pouch) and immersed in 30 mL of swelling media at 37 °C. At fixed time intervals, the beads were taken out from the medium and wiped gently with Whatman (115A) filter paper and weighed. The degree of swelling of the beads (%) with respect to time was calculated according to eq 3
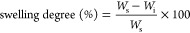
3where *W*s is the weight of the beads in the swollen state, and *W*_i_ is the initial weight of the beads.

### 2.7 Drug Incorporation and Release Studies

DCA beads (prepared *via* method II at 90 °C) were immersed in 5 mL of DCF solution at different concentrations (*c* = 1, 5, 10, 15, and 20 mg/mL, dissolved in PBS buffer at pH 7.4) for 3 h, ensuring 2 beads/cm^3^ of the solution. Following this, the beads were taken out from the DCF solution, kept on a glass plate, and dried at room temperature for 24 h.

The DCF release from the encapsulated beads was determined according to the USP paddle method (United States Pharmacopeia, 35th ed.).^57^ Twelve DCF-encapsulated DCA beads were sunk in a Sotax AT7 smart dissolution tester (SOTAX, Switzerland) in 500 mL of SBF (pH 1.2) and PBS buffer (5.5 and 7.4) at 37 °C. The released DCF amounts were measured with a UV/Vis spectrometer (PerkinElmer LAMBDA 25, Germany) at a wavelength of 276 nm, and the concentrations were calculated using calibration curves. The release experiments were carried out in triplicate.

The drug loaded in the DCA beads was determined by the following eq 4
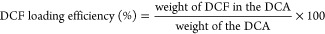
4

To evaluate the release mechanisms of DCF from the DCA-encapsulated beads, the obtained release data at different pH values and different drug concentrations were fitted with four kinetic models, namely, zero-order, first-order, Korsmeyer–Peppas, and Higuchi models, respectively.

The zero-order kinetic model can be described as shown in eq 5. The zero-order kinetics were employed to relate to the drug release where the release kinetics are concentration-dependent
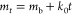
5where *m*_*t*_ is the amount of drug released in time *t*, *m*_b_ is the initial concentration of the drug in the solution before release, and *k*_0_ is the zero-order release rate constant.

First-order kinetics (eq 6) is used to describe the release of the drug where the release is concentration-dependent
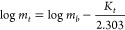
6 where *m*_t_ is the amount of the released drug in time *t*, *m*_b_ is the initial concentration of the drug and *K*_t_ is the first-order rate constant.

The Korsmeyer–Peppas kinetic model is as follows (eq 7)
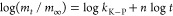
7where *m*_∞_ is the amount of drug released after an infinitive time (in our work, after 1500 min), *k*_K–P_ is the Korsmeyer–Peppas rate constant, and *n* is the diffusional exponent used to describe the drug release mechanism. In general, the value of *n* varies, depending on the shape of the drug carrier system. In spherically shaped systems, for which an *n* value of 0.45 is determined, the release mechanism is Fickian diffusion. Whereas, if 0.45 < *n* < 0.89, then a non-Fickian diffusion release, or an anomalous transport mechanism, is implied. If *n* > 0.89, then the drug release is dominated by the case II release mechanism, that is, a combination of diffusion and the swelling-controlled process.

The Higuchi kinetic model is described by eq 8 as follows 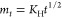
8where *m*_*t*_ is the amount of drug released at time *t* and *K* is the Higuchi constant.

### 2.8 *In Vitro* Cell Culture Studies

All bead samples were transferred into Eppendorf tubes (2 mL) and were sterilized under ultraviolet light for 30 min. After sterilization, they were transferred into the cell culture medium–advanced DMEM with 5% FBS. Before the bead samples were added to the cells, the beads were acclimatized by being incubated at 37 °C and 5% CO_2_ for 24 h (prepared according to the ISO 10993-12 material extract preparation protocol). The fibroblasts were seeded in a concentration of 10,000 cells/well in a 96-well microtiter plate. After 24 h of cell incubation, the samples were added to the cells in four repetitions. For this purpose, we used the as-prepared samples and their dilutions of 1:2, 1:4, 1:8, and 1:16 in advanced DMEM with 5% FBS. DCA beads encapsulated with different amounts of DCF (0–20 mg/mL) were also tested. After 24 h of incubation at 37 °C and 5% CO_2_, the samples’ biocompatibility/viability were assessed using the 3-(4,5-dimethylthiazolyl-2)-2,5-diphenyltetrazolium bromide (MTT) assay (Sigma-Aldrich, Germany). The purple formazan crystals formed by active cells were quantified by measuring the sample absorbance spectrophotometrically (using a Varioskan instrument, Thermo Fisher Scientific Inc., Germany) at 570 nm. The overall testing was performed following the ISO 10993-5 standard.

## 3 Results and Discussion

### 3.1 Fabrication and Characterization of the Cellulose Beads

**Figure 1:**
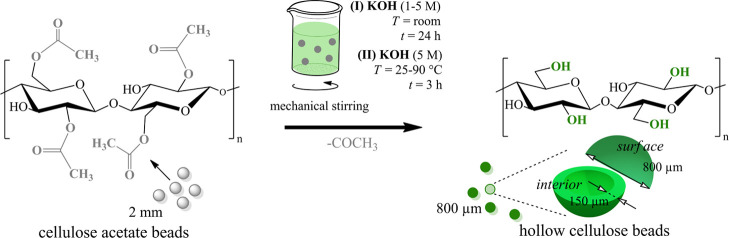
Illustration of the one-step fabrication of hollow cellulose beads from solid spherical CA beads *via* the base-catalyzed deacetylation method.

Cellulose beads with micrometer (μm) size have been used in various advanced applications ranging from chromatography, solid-support synthesis, and protein immobilization to the sustained release of targeted drugs.^58,59^ Although different starting cellulose sources, solvents, and regeneration methods have been used for the fabrication of cellulose beads, all of them result in the formation of porous beads, which is achieved by either the dropping or dispersion techniques.^53^ These techniques involve a multistep and tedious process, as reported in the literature.^34−36,53^ In the current study, we aimed to develop a simple, single-step, and water-based procedure to achieve spherical hollow cellulose as one of the most attractive alternative pH-responsive delivery systems. Cellulose beads with a micrometer-sized hollow interior, which are sufficiently porous, are particularly advantageous, as they allow a higher drug encapsulation and enable a controlled drug release. For this purpose, for the first time, we combined the use of commercially available spherical beads of solid CA and a well-known base-catalyzed deacetylation procedure to regenerate the cellulose structure from electrospun nanofibers^43^ and the spin-coated thin films of CA.^60,61^ CA beads are beneficial for drug delivery applications in many ways, as CA itself is inexpensive, biodegradable, and biocompatible, among others.^62^ Base-catalyzed deacetylation is a simple and straightforward method to convert CA directly into DCA and control its properties (*e.g.*, hydrophobicity/hydrophilicity, swelling).^43,60^ Therefore, in the current study, the spherical solid CA beads (diameter: 2 mm and density: 1.3 g/cm^3^) were transformed into hollow spherical DCA or cellulose beads, as shown in Figure 1. To obtain spherical DCA beads, CA beads were treated with different concentrations of an alkaline solution (KOH, 1 and 5 M), temperatures (25–90 °C), and times (0–24 h). These treatments resulted in the formation of DCA beads with a porous structure and a hollow interior (see Figure 3). To confirm the deacetylation of the entire bead (both surface and interior), each bead (treated/untreated) was cut in half carefully and used for further analysis. The fully deacetylated hollow cellulose beads were used for the encapsulation and controlled release of the drug DCF to demonstrate their potential application as an advanced drug delivery system (*e.g.*, targeted, sustained drug delivery).

#### 3.1.1 Structure and Composition

**Figure 2:**
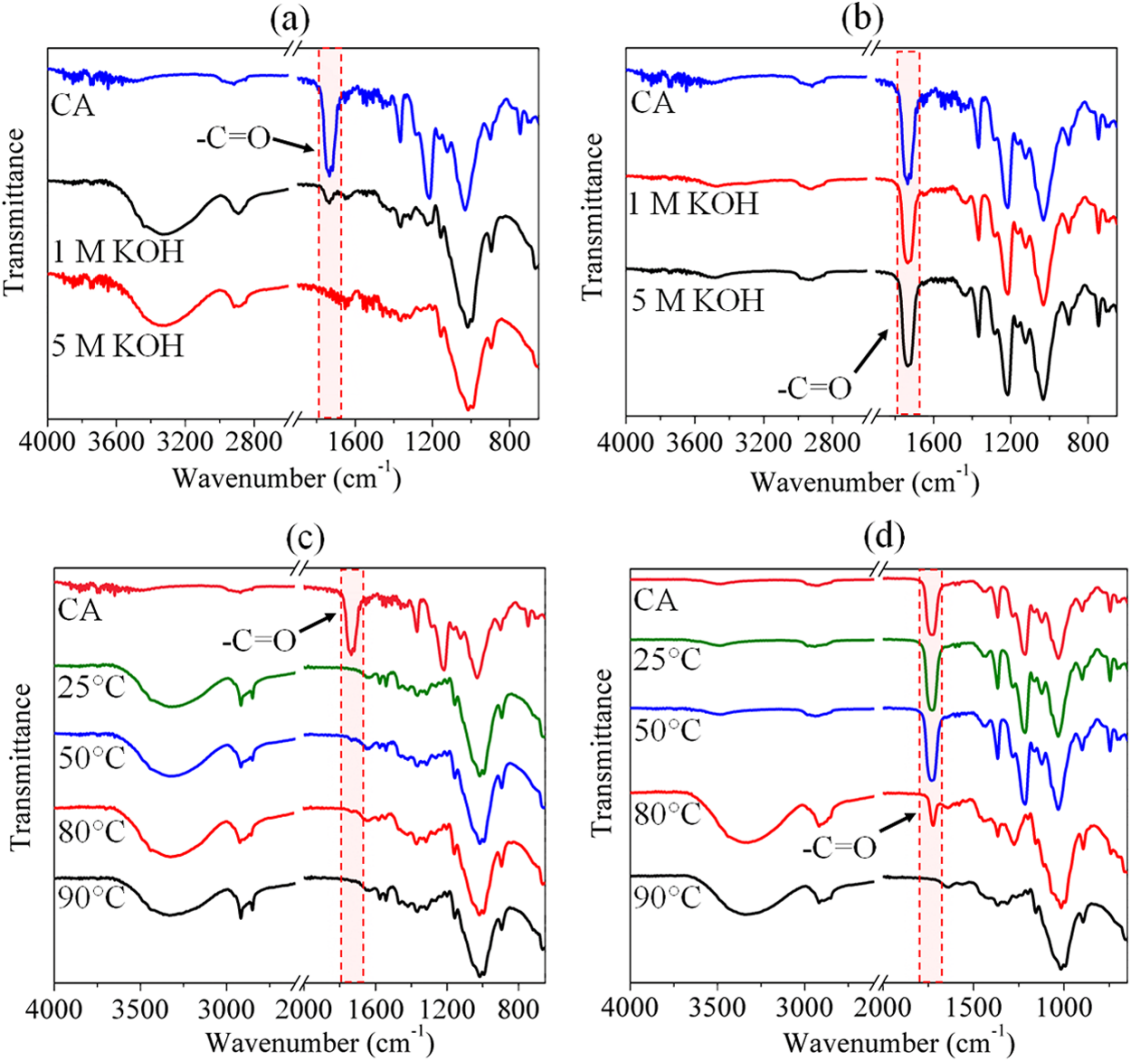
ATR–FTIR spectra of CA beads (a,c: surface; b,d: interior) before and after treatment with different concentrations of potassium hydroxide solutions (KOH) at an ambient temperature (25 °C, a,b) and with a 5 M KOH solution at different temperatures (25–90 °C, c,d) for 3 h.

The ATR–FTIR spectra of CA beads treated with KOH solution at different concentrations and at room temperature (25 °C, according to method I) are shown in Figure 2 (a: surface and b: interior). The characteristic peaks of untreated CA were observed at 1730 cm^–1^ (−C=O), 1458 cm^–1^ (O=C–OR), 1365 cm^–1^ (−CH_2_), 1030 cm^–1^ (C–O–C), and 902 cm^–1^ (−C–H), with a minor contribution of an −OH peak at 3328 cm^–1^.^60,61,63^ At 1 M KOH (Figure 2a), the emergence of a broad and intense −OH peak was evident. In addition, the intensity of the characteristic ester carbonyl peak at 1730 cm^–1^ decreased drastically along with the other characteristic peaks of CA. At a higher KOH concentration (5 M), the carbonyl peak disappeared, indicating that the surface of the beads was completely deacetylated. However, in the interior of the beads (Figure 2b), neither the emergence of a broad −OH peak nor the reduction of the carbonyl peak was observed at both KOH concentrations. This confirms that the bead’s interior was not deacetylated. The DS (or acetylation) of the CA beads before and after deacetylation (see Figure S2a,b, Supporting Information) was estimated using the ratio between the acetyl C=O stretching of the ester peak at 1730 cm^–1^ and the C–O stretching of the cellulose backbone at 1030 cm^–1^, as described by El Nemr *et al.*(56) A DS of 2.85 was determined for the entire untreated CA beads (surface and interior). After treatment with 1 M KOH, DS at the surface was decreased to 0.4. However, no decrease in DS was observed inside the beads, regardless of the KOH concentration used. We could confirm that the surface properties of the beads, such as hydrophobic/hydrophilic properties, can be tailored by varying the concentration of the base (KOH) during deacetylation.

To achieve complete deacetylation of the CA beads, the latter were treated with 5 M KOH at increasing temperatures, from 25 to 90 °C (see Section 2.2, method II). The ATR–FTIR spectra obtained from these experiments are shown in Figure 2c,d. The impact of temperature on the deacetylation of the beads can be seen clearly. As expected, no peak of the carbonyl group at 1730 cm^–1^ was detected at the surface of the beads, regardless of the temperature (Figure 2c), confirming the complete deacetylation (decrease of DS from 2.85 to 0) of the surface of the beads. On the contrary, a general trend of decrease in the intensity of the carbonyl group-related peak at 1730 cm^–1^ with increasing temperature was observed for the bead’s interior (Figure 2d). In parallel, the emergence of a broad −OH peak at 3328 cm^–1^ and other characteristic peaks of cellulose in the fingerprint region (between 800 and 1400 cm^–1^)^43,64,65^ was also observed. This effect was even more pronounced at 80 °C, where DS decreased from 2.85 to 0.94 (see Figure S2c,d, Supporting Information). No carbonyl peak was observed at 90 °C, and the spectra resembled the structure of neat cellulose,^43,60,61^ indicating that the CA beads had been completely transformed into cellulose beads. Overall, compared to method I, method II allows the control of the hydrophilic/hydrophobic character and DS of the entire bead structure by simply varying the conditions of the base catalysis (*e.g.*, temperature). This type of character is desired if beads are to act as carriers in targeted drug delivery. They can be used selectively to encapsulate hydrophobic or hydrophilic drugs, depending on the application.^34,36,66^

#### 3.1.2 Bead Morphology

**Figure 3:**
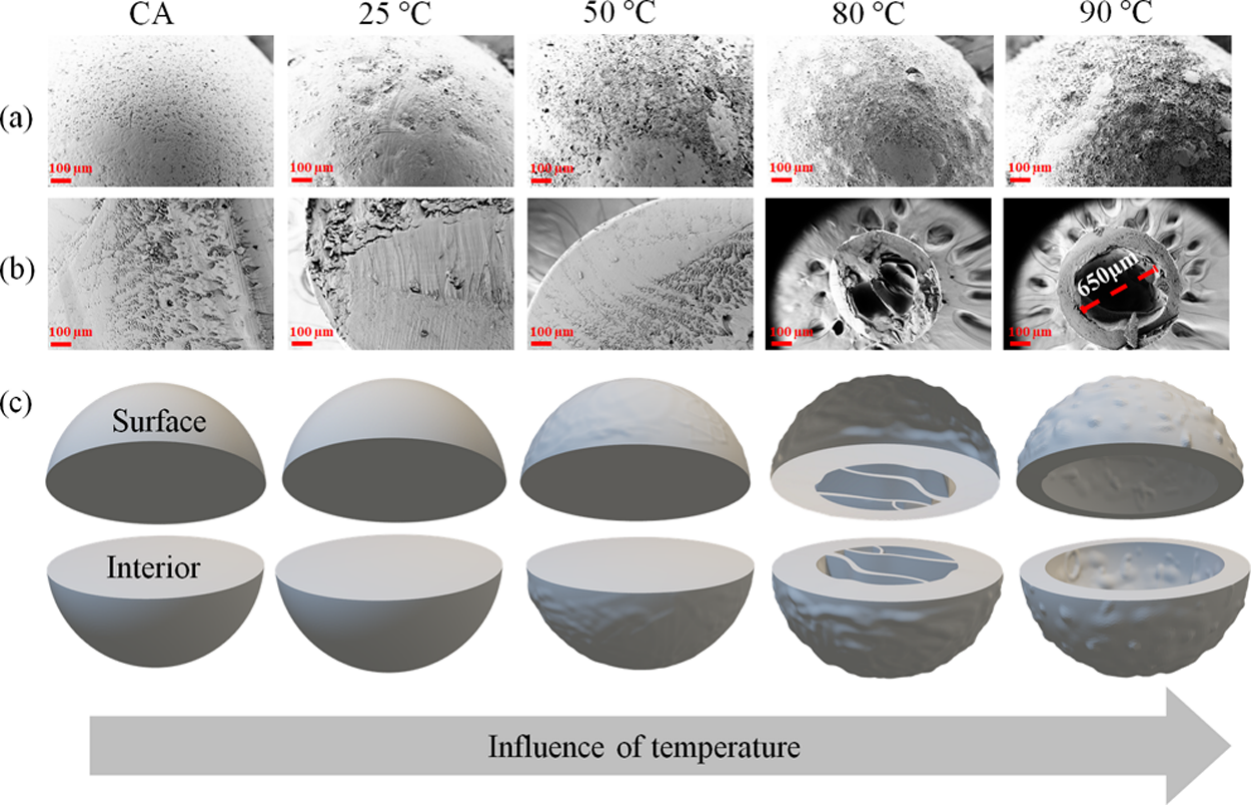
SEM images of CA beads (a: surface and b: interior) before and after treatment with 5 M potassium hydroxide solution (KOH) at different temperatures. The size of the scale bar is 100 μm. (c) Schematic models are shown to illustrate the SEM results better due to changes in both the surface and interior of the CA beads as a function of temperature.

The SEM morphology (a: surface and b: interior) of CA beads before and after treatment with 5 M KOH at different temperatures is depicted in Figure 3a–c. In general, the surface (skin, Figure 3a) of the untreated CA beads is smoother and has fewer pores. The treated beads exhibit a rougher and more porous morphology. This behavior was more pronounced with the increasing temperatures. The calculated pore size for all samples was in the range of *ca.* 10 μm (according to the SEM data). Although the direct measurement of roughness on the surface of the beads is not possible, it was evident that the surface became rougher with incremental temperature. It is suggested that some parts of the base material and/or the acetyl (−COCH_3_) groups are removed from the surface as the result of deacetylation at a high temperature and exposure to a strong base (5 M KOH). This led to changes in the surface morphology and porous structure. This is in good agreement with the ATR–FTIR spectroscopy results (Figure 2a), which showed the disappearance of the acetyl groups with increasing temperature.

As can be seen in Figure 3b, densely packed solid structures are observed in the interior of the untreated CA beads. When compared to the latter, no major changes in structure and morphology are noticed till 50 °C, suggesting that no major mass loss or removal of acetyl groups has occurred at the interior of the beads at this temperature. This can be correlated with the ATR–FTIR results, where the intensity of the acetyl peaks remained the same as in the untreated CA beads treated with temperatures up to 50 °C. This further indicates that the interior of the beads is less accessible at low temperatures, and the diffusion of KOH is therefore limited as well (considering the densely packed CA bead structure). Surprisingly, at higher temperatures (80–90 °C), some material was removed (even from the interior, besides the observable changes in surface porosity and morphology occurring at these temperatures). Especially at 90 °C, a hollow structure with a wall thickness of 150 μm (see Figure S1, Supporting Information) and an inner diameter of 650 μm was formed, implying that the combination of higher temperature and strong base caused extensive deacetylation, leading to the formation of the hollow structure. We can show that a single-step deacetylation treatment at high temperature is useful to transform the spherical solid systems of acetylated polymers into deacetylated spherical systems with partially or completely hollow interiors. Such systems with hollow interiors and porous surface structures may be advantageous as drug carriers in medicine. For example, they can be used to enhance the encapsulation capacity of drugs such as DCF and/or to enable a continuous and simultaneous fluid exchange with the environment, as in the case of drug release.^9^

**Figure 4:**
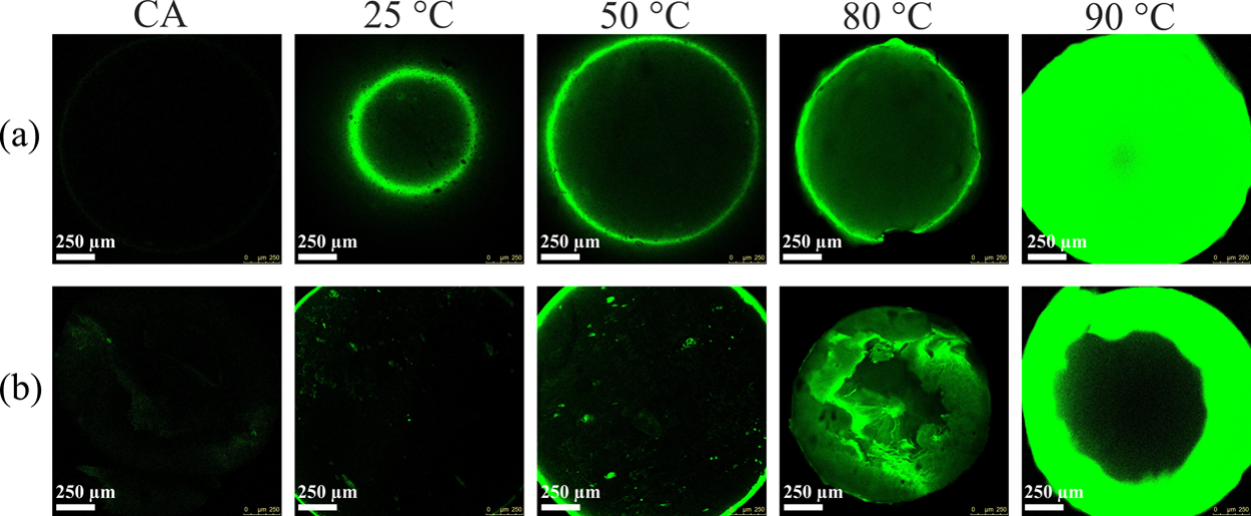
CLSM images of CA beads (a: surface and b: interior) before and after treatment with 5 M potassium hydroxide solution (KOH) at different temperatures.

To confirm the completion of deacetylation of the entire structure of CA beads further, we used a combination of staining and confocal laser scanning microscopy (CLSM)-based methods. Figure 4 shows the CLSM images (a: surface and b: interior) of untreated and treated CA beads (5 M KOH, 25–90 °C) stained with the DTAF dye. No fluorescence signal was detected on the surface as well as in the interior of the untreated CA beads, as the latter are nonreactive and cannot be labeled by DTAF; thus, the nonspecific binding of DTAF was low. On the other hand, bright and strong fluorescence intensity was observed at the periphery of the treated beads. The intensity of the fluorescence signal increased with the increasing temperature (25–90 °C), which is an indication that CA is transformed to cellulose gradually, due to deacetylation.^43,60,61^ This is also in accordance with the ATR–FTIR results, where the emergence of reactive −OH groups at 3328 cm^–1^ was more pronounced with the increasing temperature. At 90 °C, complete deacetylation of the surface and interior of the beads was achieved, which was evidenced by their strong and high fluorescence intensity. This confirms that the unreactive hydrophobic CA beads were completely transformed into reactive and hydrophilic cellulose.^60,61^ The ATR–FTIR spectroscopy results support this finding further, where all acetyl groups disappeared and only peaks related to the neat cellulose structure were observed.

#### 3.1.3 Mass Loss and Fluid Uptake

**Figure 5:**
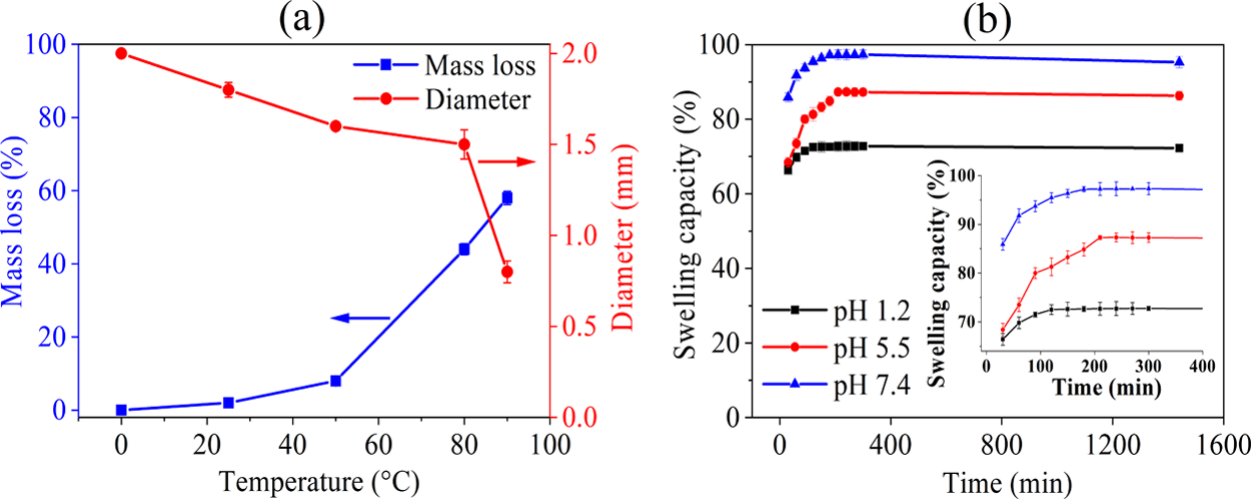
(a) Mass loss and change in diameter of CA beads treated with 5 M KOH at different temperatures and (b) swelling capacity of DCA beads (90 °C, method II) in different pH environments as a function of time.

The influence of temperature (25–90 °C) on the mass loss (in percent, %) and size (in mm) of CA beads after treatment with 5 M KOH is shown in Figure 5a. Up to 50 °C, the mass loss was observed within a range of up to 10%. Considering the absence of carbonyl peaks at 1730 cm^–1^ (Figure 2) in the interior of the beads, it can be assumed that the observed mass loss of 10% only occurred at the surface of the beads and not from the interior, which is in good agreement with the CLSM results (see Figure 4). At higher temperatures (80–90 °C), the mass loss increased from 40 to 60%, which can be attributed to the removal of the acetyl groups. This is also evident in the size of the beads, where the original bead size of untreated CA decreased from 2 mm to 800 μm with increasing temperature. It is suggested that the base-catalyzed treatment (*i.e.,* higher temperature: 90 °C and KOH concentration: 5 M) resulted in considerable mass loss and shape changes. This can be correlated with the ATR–FTIR data, where the actual structural transformation of the base CA beads was observed. Interestingly, although the beads shrank in size, they did not lose their spherical shape. The 800 μm size of the fully deacetylated CA beads remains comparable to the bead size of other drug delivery systems such as chitosan,^34^ carboxymethyl cellulose/chitosan,^67^ alginate/chitosan,^68^ alginate/carboxymethyl cellulose,^69^ pectin,^70^ TEMPO-oxidized cellulose,^35^ and so forth.

Swelling is one of the most important parameters that determine the diffusion and release rate of encapsulated drugs in different fluid environments and, as such, affects the overall drug delivery mechanism importantly. The extent of swelling is used to determine the ability of drug carriers to absorb water, which can be analyzed quantitatively by the weight gain of the beads. Among others, the swelling degree can be influenced by two main factors: (i) pH and (ii) time.^71^ To determine the swelling/fluid uptake capacity, hollow DCA beads prepared at 90 °C were immersed for an extended period (24 h) in swelling media prepared at three different pH values (1.2, 5.5, and 7.4), corresponding to the gastric^35^ and cancer^72^ environments, as well as the values during wound healing or tissue regeneration.^34^ The results of the swelling measurements are shown in Figure 5b. In general, the swelling capacity increases with time at all pH values. At pH 1.2, the beads swelled quickly within a few minutes and reached a plateau after 150 min compared to the other two pH values. However, the total swelling capacity of the beads decreased with lower pH ranges, in the following order: pH 1.2 < pH 5.5 < pH 7.4. This can be related to the degree of ionization of the −OH groups of DCA beads, which is lower at pH 1.2 and higher at the other two pH values tested. The pH-responsive fluid uptake capacity of DCA beads is useful to fine-tune the encapsulation amount and release rate of drugs that exhibit pH-dependent solubility behavior for targeting specific tissues/organs, where the change in pH can be used to control drug release further and, hence, its therapeutic effect.

### 3.2 *In Vitro* pH-Responsive Drug Release

**Figure 6:**
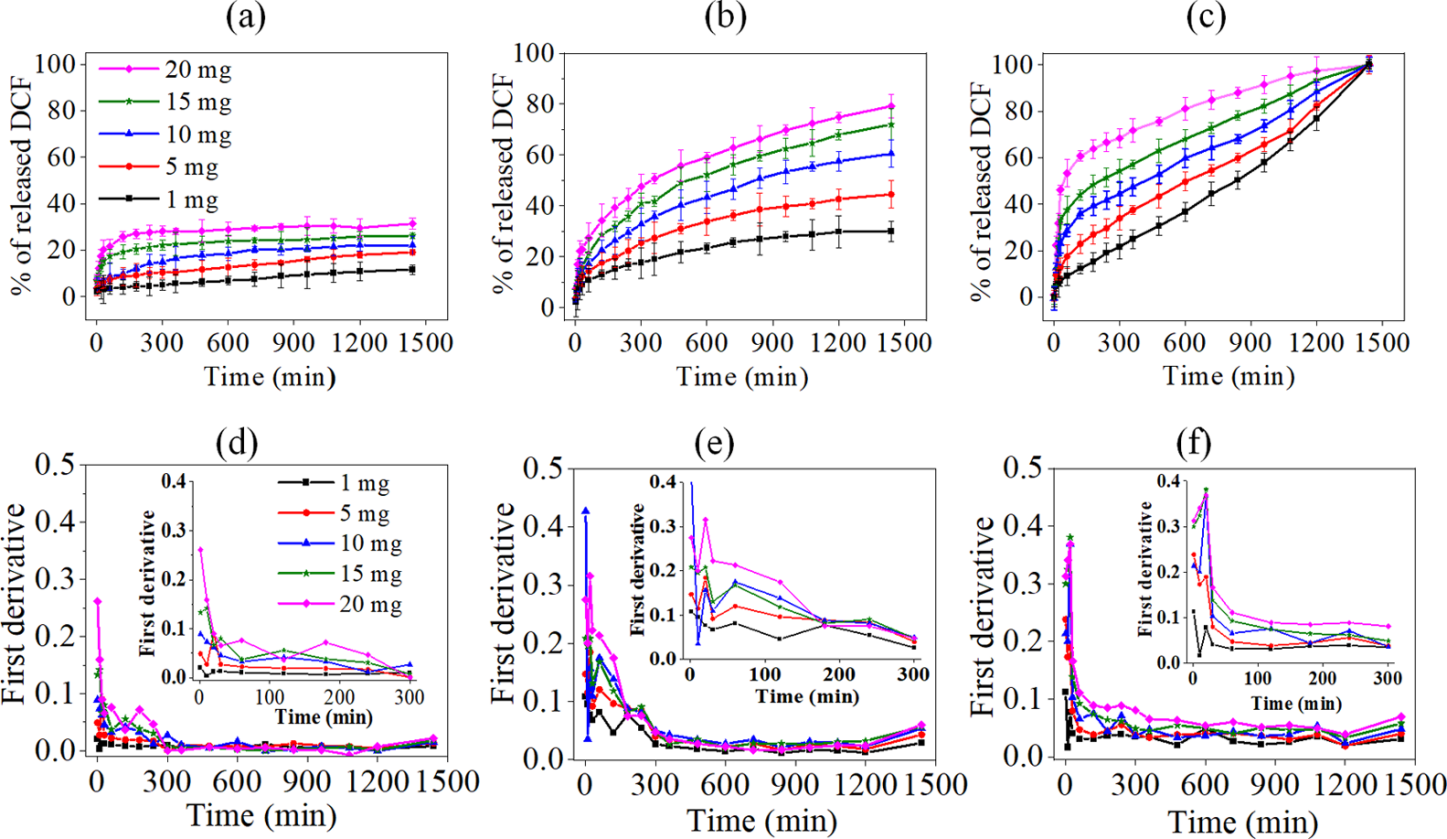
Release profiles (top) and first derivatives (bottom) of DCF in simulated body fluid (pH 1.2: a,d), in PBS (pH 5.5: b,e), and in PBS (pH 7.4: c,f).

**Figure 7:**
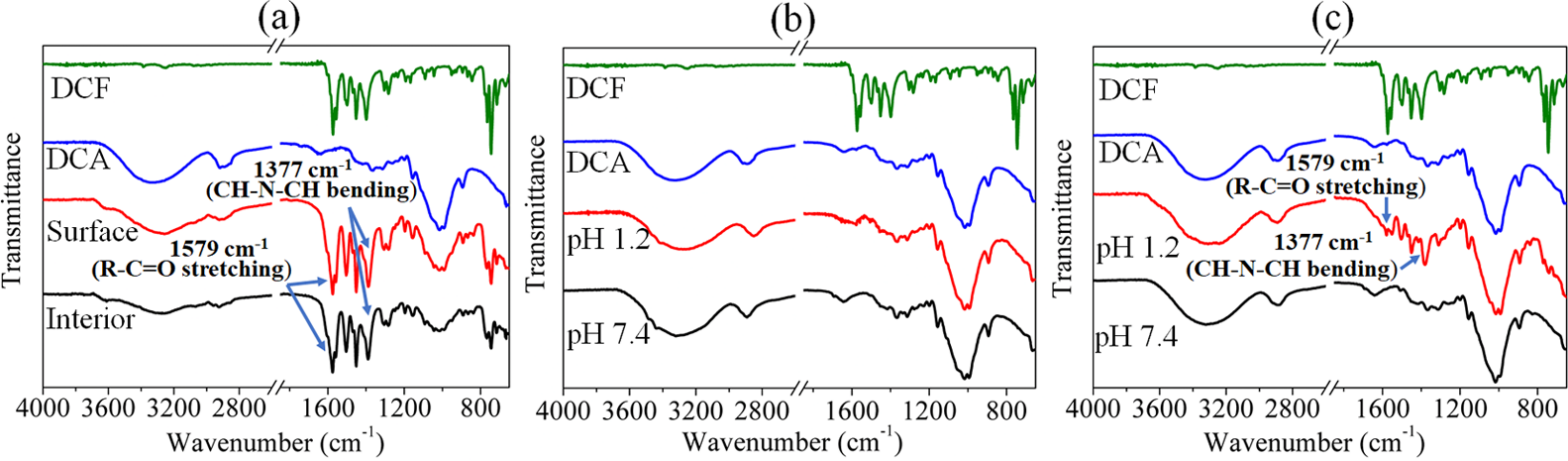
ATR–FTIR spectra of DCF (20 mg/mL)-encapsulated DCA beads before and after *in vitro* pH-dependent release. (a) Before release: DCF-encapsulated DCA beads; (b,c) after release: (b) surface and (c) interior.

A good drug carrier/release system is anticipated to retain an adequate amount of drug and often to have a pH-responsive behavior (*e.g.*, to make use of pH changes to control drug release in different pathophysiological conditions). Therefore, we investigated the release properties of DCF-encapsulated DCA beads in different pH environments. This was done to simulate the situation and to understand the release kinetics of DCF/DCA beads in various pH environments, including the gastrointestinal tract (pH 1.2),^35^ tumor microenvironment (pH 5.5),^72^ and (soft and hard) tissue engineering (pH 7.4).^34^ The nonsteroidal anti-inflammatory DCF was chosen as a model drug, for its pH-dependent solubility, to aid investigations in the encapsulation and release efficiency studies of the hollow DCA beads. The release profiles of DCF at different pH values (a: pH 2.0, b: pH 5.5, and c: pH 7.4) and at different drug loading concentrations (DCF: 1–20 mg/mL) at increasing durations of time are shown in Figure 7. The DCF loading content in the DCA bead interior is regardless of the loading concentration in the range of *ca.* 20–85% (1–25 mg/mL). In particular, the DCF loading content in the bead was in the following order: 19.8% (1 mg) < 28.3% (5 mg) < 35% (10 mg) < 45.6% (15 mg) < 60.2% (15 mg) < 71% (20 mg) < 85.3% (25 mg). This is comparable to the values obtained with other drug loading systems, such as cellulose nanocrystals/polyvinyl alcohol (PVA),^35^ CA/PVA, carbon dot/alginate, iron oxide/PVA,^73^ polyethylene glycol-based polyprodrug amphiphiles,^74^ and so forth. Although DCF is highly water-soluble (21.3 g/L), the release profiles of DCF from all three media studied (*i.e.,* pH 1.2–7.4) are nevertheless very different (Figure 6a–c), indicating pH-responsive release characteristics.

At pH 1.2, the release curves at all concentrations showed a biphasic behavior, meaning that an initial fast release (burst effect, first part) is accompanied by a prolonged/sustained release (second part). These biphasic release patterns are also seen at pH 5.5 and 7.4. At all pH ranges tested, the burst release was noticed up to 30 min and at all concentrations of DCF. At pH 1.2, 3–11% of DCF was released during the first part when the DCF concentration increased from 1 to 20 mg/mL. At pH 5.5 and pH 7.4, the maximum observed release of DCF was 10–30 and 10–60% during the initial burst release. An explanation for the observed burst effect is the release of drugs into the surrounding media, which were adsorbed heterogeneously on the surfaces of the beads during encapsulation, keeping the surface of the pores open for subsequent diffusion of the drugs.^75^ At pH 1.2, a slow release (25%) was observed for up to 300 min following the burst effect. The second release phase was the so-called “plateau,” which is due to the diffusion-controlled release of the remaining drug in the bead. In the other two cases, a rapid release of the drug continued after the burst release, increasing maximally for all DCF concentrations up to 300 min, followed by a slower and prolonged release for the duration of the next 19 h.

In all cases, the total release of the drug increased with increasing DCF concentrations. The amounts of DCF released from the beads increased significantly as the pH of the release medium increased from acidic to neutral. It can be stated that the release pattern and amounts of the drug can be fine-tuned by simply increasing the drug concentration or by altering the pH of the release medium. The maximum amount of drug released at pH 1.2 was *ca.* 30% at the highest concentration (20 mg) of the drug after 24 h, whereas at pH 5.5 and pH 7.4, the release of the drug was increased to 80 and 100% at the same concentration. The release of the drug is due to the diffusion of water into the porous structure of the beads, resulting in swelling. This may cause the incorporated drug to dissolve, diffuse out of the beads, and, subsequently, be released into the surrounding medium. It is expected that the swollen DCA beads will release higher amounts of the drugs to the medium. From the results of the swelling experiments, it can be stated that the less swollen DCA beads at pH 1.2 released lower amounts of DCF than the other two pH values at which maximum swelling of the beads was observed. DCF is a weak acid with a p*K*_a_ value of 4.0, is weakly soluble in acidic solutions, and dissolves readily in intestinal fluid and water.^76^ Therefore, the lower solubility of DCF at pH 1.2 limits the initial release of drug from the surface and hollow interior of the beads and, hence, the overall release of DCF. Increasing the pH to 5.5 and pH 7.4 caused DCF to dissolve readily due to ionization, ultimately resulting in greater solubility and a higher release profile (Figure 6b,c).

To understand the kinetics and mechanism of DCF released at different pH values further, the experimental data in Figure 6a–c were fitted by linear regression analysis with different kinetic models, including zero-order, first-order, Higuchi, and Korsmeyer–Peppas models. Even though different kinetic models were considered, the model with the highest square of the correlation coefficient (*r*^2^, see Table 1) was found to be the best fit for the release data shown in Figure 6a–c.

**Table 1:** *r*^2^ Values Obtained from the Linear Fitting on Both Regions (Part I: Fast Release and Part II: Slow Release) of All DCF-Coated Samples at pH 1.2–7.4 Using Different Kinetic Models

	kinetic model									
DCF	zero-order		first-order		Higuchi		Korsmeyer–Peppas			
*c* (mg)	1st part	2nd part	1st part	2nd part	1st part	2nd part	1st part	*n*	2nd part	*n*
	pH 2									
1	**0.99±0.1**	**0.99±0.12**	0.98 ± 0.11	0.97 ± 0.1	0.98 ± 0.13	0.98 ± 0.14	0.76 ± 0.2	0.41 ± 0.02	0.97 ± 0.12	0.29 ± 0.01
5	**0.98±0.12**	**0.99±0.1**	0.97 ± 0.11	0.97 ± 0.1	0.97 ± 0.11	0.98 ± 0.16	0.85 ± 0.1	0.38 ± 0.1	0.97 ± 0.11	0.52 ± 0.01
10	**0.98±0.12**	**0.99±0.12**	0.97 ± 0.12	0.88 ± 0.14	0.97 ± 0.14	0.79 ± 0.09	0.86 ± 0.14	0.22 ± 0.05	0.92 ± 0.14	0.56 ± 0.04
15	**0.99±0.1**	**0.99±0.08**	0.97 ± 0.2	0.95 ± 0.13	0.97 ± 0.12	0.96 ± 0.04	0.88 ± 0.16	0.34 ± 0.1	0.96 ± 0.11	0.43 ± 0.01
20	**0.99±0.2**	**0.99±0.12**	0.98 ± 0.1	0.81 ± 0.2	0.97 ± 0.11	0.88 ± 0.11	0.91 ± 0.09	0.41 ± 0.02	0.97 ± 0.12	0.38± 0.01
	pH 5.5									
1	0.86 ± 0.12	0.98 ± 0.17	0.97 ± 0.12	0.98 ± 0.01	**0.98±0.2**	**0.99±0.2**	0.97 ± 0.12	0.44 ± 0.01	0.98 ± 0.12	0.55 ± 0.1
5	0.84 ± 0.11	0.97 ± 0.5	0.98 ± 0.33	0.97± 0.13	**0.99±0.15**	**0.99±0.18**	0.98 ± 0.14	0.46 ± 0.04	0.97 ± 0.9	0.45 ± 0.09
10	0.90 ± 0.09	0.97 ± 0.12	0.98 ± 0.14	0.98± 0.14	**0.99±0.13**	**0.99±0.16**	0.98 ± 0.09	0.77 ± 0.1	0.98 ± 0.18	0.34 ± 0.17
15	0.88 ± 0.14	0.97 ± 0.14	0.98 ± 0.17	0.98± 0.15	**0.99±0.11**	**0.99±0.11**	0.98 ± 0.17	0.45 ± 0.14	0.98 ± 0.12	0.73 ± 0.07
20	0.89 ± 0.11	0.96 ± 0.11	0.98 ± 0.15	0.98 ± 0.2	**0.99±0.12**	**0.99±0.12**	0.98 ± 0.11	0.88 ± 0.07	0.98 ± 0.5	0.48 ± 0.1
	pH 7.4									
1	0.99 ± 0.14	0.98 ± 0.2	0.97 ± 0.2	0.96 ± 0.2	0.96 ± 0.17	0.98 ± 0.14	**0.99±0.01**	**0.98±0.14**	**0.99±0.17**	**1.04±0.12**
5	0.94 ± 0.2	0.98 ± 0.14	0.95 ± 0.21	0.97 ± 0.18	0.99 ± 0.21	0.98 ± 0.13	**0.99±0.14**	**1.01±0.21**	**0.99±0.18**	**1.02±0.15**
10	0.94 ± 0.14	0.98 ± 0.14	0.95 ± 0.19	0.98 ± 0.21	0.98 ± 0.1	0.98 ± 0.11	**0.99±0.11**	**0.99±0.14**	**0.99±0.19**	**1.03±0.17**
15	0.92 ± 0.17	0.98 ± 0.19	0.95 ± 0.16	0.99 ± 0.13	0.98± 0.15	0.98 ± 0.09	**0.99±0.13**	**1.11±0.14**	**0.99±0.21**	**1.12±0.19**
20	0.9 5 ± 0.021	0.98 ± 0.1	0.99 ± 0.1	0.98 ± 0.1	0.98 ± 0.2	0.98 ± 0.1	**0.98±0.1**	**1.10±0.3**	**0.99±0.1**	**0.99±0.1**

Even though the release curves look relatively simple (Figure 6a–c), the entire release curve (*i.e.*, 0–1500 min) could not be fitted satisfactorily with any of the models used in this study. Therefore, we divided the release curves for all samples and all pH values into two parts (part I and part II). Part I refers to the initial fast release of 0–300 min, while the prolonged release, that is, 300–1500 min, was set as the second part. The division of each release curve into two parts can be verified further by calculating the first derivative (Figure 6d–f), which shows two release regions and a division point at 300 min of the release curve. From Table 1 and Figure S3 (see the Supporting Information), it can be seen that, at each pH, despite the different models employed for the best fit, only one model can describe the release mechanism fully and satisfactorily. Considering the best fit (highest *r*^2^ value in Table 1), as well as the calculated results of the first derivative (Figure 6d–f) based on the release data (Figure 6a–c), it can be assumed that more than one mechanism is involved in the release of the drug from the DCF-loaded DCA beads.^77^ At pH 1.2, the best fit with maximum *r*^2^ values was obtained for the zero-order model for both parts of the release curve compared to the other kinetic models. This indicates that the release rate of DCF is independent of its concentration. Such a release rate is particularly desirable for a certain class of medicines, where a continuous therapy is desired without potential changes in drug release. Such a clinical setting is, for example, in the case of pain relief.^8,9,67^ At pH 5.5, the Higuchi model showed the best fit with a maximum *r*^2^ value, confirming the *t*^1/2^ dependence of the drug release, which is a pure feature of the Fickian diffusion mechanism, implying that drug release is controlled predominantly by diffusion. On the other hand, it cannot be excluded that the drug is also released by non-Fickian diffusion, as the DCA beads are porous and exhibit a hollow interior. This may result in maximum simultaneous and rapid release of the drug from both the surface and bulk parts of the beads.^78^

At the physiological pH (7.4), the best fit with maximum *r*^2^ values was obtained for the Korsmeyer–Peppas kinetic model, which best describes the drug transport mechanism where the value of diffusion coefficient “*n*” was calculated by the slope of the straight line of the data. This model is the most common to describe the kinetics of drug release from polymeric systems when the release mechanism is unknown or more than one release phenomenon is involved.^79^Table 1 shows that the absolute value of “*n*” is greater than 0.9 for DCF concentrations, indicating a nearly anomalous or non-Fickian case II transport mechanism, where Fickian is the transport of drug through the pores of DCA beads, and anomalous shows the transport mechanism of the drug by a combined effect of diffusion and swelling.^72^ The release of DCF is not only attributed solely to a “simple” diffusion-controlled mechanism but is often accompanied additionally by a combination of swelling and subsequent erosion of the drugs adsorbed on the surface when they come into contact with a buffer solution.^80^ In the swelling test performed at pH 7.4, where the beads were highly swollen, the release of drug is clearly of the Fickian type, that is, diffusion-controlled type, as indicated by the highest *r*^2^ value and the exponent “*n* < 0.45” value. As the first-order model resulted in much lower *r*^2^ values, it was not considered for further interpretation and discussion of the results.

#### 3.2.1 Structure and Composition before and after *In Vitro* pH-Dependent Release

We also verified the encapsulation of DCF in the whole bead structure before and after the *in vitro* release studies by infrared spectroscopy (Figure 7). The spectra of DCF, DCA, and DCF-encapsulated beads before *in vitro* release are shown in Figure 7a. For clarity, only the results of DCF at a concentration of 20 mg/mL are depicted, and the results obtained at pH 5.5 are not shown. In the DCF-encapsulated system, besides the peaks of DCA, the characteristic peaks of DCF functional groups are observed at 3253 cm^–1^ (−NH stretching of the secondary amine), 1571 cm^–1^ (−C=O stretching of the carboxyl ion), 1500 cm^–1^ (−C=C ring stretching), 1450 cm^–1^ (−CH_2_ bending), 945 cm^–1^ (−C–O–C stretching), and at 746 cm^–1^ (−C–Cl stretching), both on the surface and interior of the bead. After *in vitro* release at pH 1.2, 7.4 (Figure 7b, surface), and pH 5.5 (data not shown), peaks characteristic of DCF functional groups were no longer observed on the surface of the beads, indicating that all surface-coated drugs were released into the medium during the *in vitro* release. While peaks characteristic of DCF functional groups were still detectable after release at pH 1.2 and pH 5.5 (data not shown), no such characteristic peaks were observed in the interior at pH 7.4. These results agree well with the release studies in which fewer amounts of the drug are released at acidic pH than at neutral pH. This may be due to the low solubility of DCF at acidic pH and increased solubility at pH 7.4, as mentioned above. It is clear that all the encapsulated drug molecules in the interior diffused successfully through the pores of the beads and were released into the surrounding medium at pH 7.4, whereas at pH 1.2 and pH 5.5, the unreleased drug molecules remained in the interior of the structure of the beads during release. Overall, ATR–FTIR spectroscopy is a useful tool and sensitive enough to detect the successfully encapsulated and the unreleased part of DCF from the carrier matrices.

### 3.3 Biocompatibility Test

**Figure 8:**
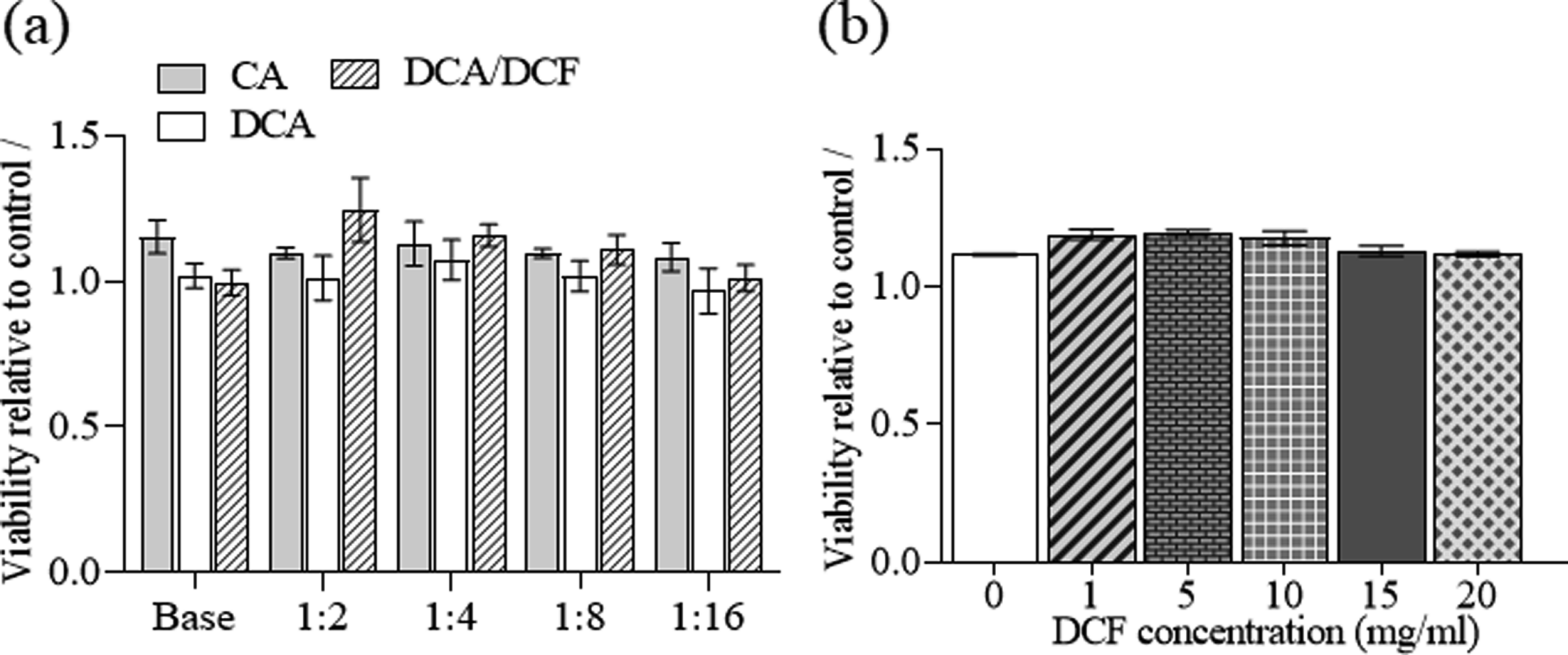
Fibroblast biocompatibility of CA, DCA, and DCF-encapsulated DCA bead-based MTT assays (a) at different dilution series and (b) at different concentrations of DCF (control sample was DCA: 0 mg/mL DCF). The shown viabilities are presented as average (based on at least four replicates).

Biocompatibility is a prerequisite for any new drug delivery system to be used for either type of application in humans. This is also true for the targeted applications in the case of the DCA beads developed herein, for example, cancer therapy, in wound healing, and/or in boosting tissue regeneration, where pH-responsive drug delivery might improve the treatment outcomes. This study aimed to evaluate the biocompatibility of the untreated CA, DCA, and DCF-encapsulated DCA beads with human fibroblast cells, which are commonly applied for such purposes.^79,81^Figure 8a shows the influence of the beads (untreated CA and DCA) encapsulated with and without DCF on the cell viability of human fibroblasts (at different dilutions of the base material extract). In general, no considerable reduction in cell viability was observed for either the undiluted or diluted form compared to the control sample. Surprisingly, both untreated CA and DCA or DCF-encapsulated beads did not reduce or increase cell viability. This effect remained persistent with a serial decrease in cell density. This indicates that both the drug-free and encapsulated samples are biocompatible without affecting cell viability. We also tested the cell viability of DCA beads encapsulated with different amounts of DCF (1–20 mg/mL, Figure 8b). Different concentrations of DCF had no effects on cell viability compared to the control sample (DCA, 0 mg/mL DCF), indicating that the DCA beads are biocompatible regardless of the encapsulated DCF amount (even for the highest loading concentration of 20 mg/mL). This noncytotoxic behavior of DCF-encapsulated beads shows that they may be of interest for applications requiring higher amounts or different amounts of DCF and its prolonged release in different pH environments. Furthermore, these results also show that therapy individualization (regarding the dosing regimen for respective patients) can be performed safely.

## 4 Conclusions

In this work, cellulose beads with a porous surface and hollow interior were fabricated from solid spherical CA beads for the first time. This was achieved by single-step deacetylation treatment at different temperatures and varying concentrations of base (KOH). This simple aqueous and temperature-dependent treatment resulted in deacetylated cellulose beads (DCA) with adjustable DS (acetylation), wall thickness, mass, and hydrophobic/hydrophilic properties, as proven by scanning electron microscopy, infrared spectroscopy, and thickness and mass loss measurements. The fluid uptake properties of the fully deacetylated CA hollow beads (size: 800 μm) increased with the increasing pH (1.2–7.4) of the solutions, as demonstrated by the swelling studies. Encapsulation efficiency of 20–85% was achieved with increasing the DCF concentrations (1–20 mg/mL). *In vitro* release studies showed a pH-dependent release behavior, where the amount of drug released was lower at acidic pH and increased with increasing pH of the release medium. The kinetics of drug release at different DCF concentrations followed the same pattern, with a burst release during the 30 min release period, followed by prolonged release. Among the four kinetic models chosen for data analysis, the zero-order model showed the best *r*^2^ values at pH 1.2, while at the values of pH 5.5 and pH 7.4, the best *r*^2^ values are obtained for the Higuchi and Korsmeyer–Peppas models, indicating that the release of the drug is controlled predominantly by swelling and diffusion. The results of biocompatibility with human fibroblasts proved that not all the investigated beads encapsulated with and without DCF at different concentrations exhibited cytotoxicity and were, therefore, biocompatible. Considering the one-step aqueous method to prepare hollow cellulose beads and their potential use for high encapsulation efficiency and pH-responsive release behavior, it can be concluded that the biocompatible hollow cellulose beads have great potential for various biomedical applications (*e.g.,* pH-responsive drug delivery, cancer therapy, or wound healing).

## Supporting Information Available

SEM images of CA beads treated using 5 M KOH at 90 °C, ATR–FTIR spectra with the DS of cellulose beads before and after deacetylation; and drug release data of DCF-encapsulated DCA beads fitted to various kinetic models (PDF)
