# Supplementary material for: One-Step Fabrication of Hollow Spherical Cellulose Beads: Application in pH-Responsive Therapeutic Delivery
Source: ACS Appl Mater Interfaces. 2022 Jan 11;14(3):3726–39. doi: 10.1021/acsami.1c19577 (PMC8796171; doi:10.1021/acsami.1c19577)
Supplement: Supplementary file 1 — am1c19577_si_001.pdf [file am1c19577_si_001.pdf]

Supporting information

One-step Fabrication of Hollow Spherical Cellulose Beads: Application in pH-Responsive Therapeutic Delivery

Tamilselvan Mohan[†,*], Urban Ajdnik[‡], Chandran Nagaraj[€], Florian Lackner[†], Andreja Dobaj Štiglic[‡], Thirvengadam Palani[β], Lunjakorn Amornkitbamrung[§], Lidija Gradišnik[£], Uroš Maver[£], Rupert Kargl[†], Karin Stana Kleinschek[†]

[†]Graz University of Technology, Institute for Chemistry and Technology of Biobased Systems (IBioSys), Stremayrgasse 9, 8010 Graz, Austria.

[‡] University of Maribor, Faculty of Mechanical Engineering, Institute of Engineering Materials and Design, Smetanova 17, 2000 Maribor, Slovenia.

[€]Ludwig Boltzmann Institute for Lung Vascular Research, Stiftingtalstrasse 24, 8010 Graz, Austria.

[β]Shanghai Jiao Tong University, School of Chemistry and Chemical Engineering and State Key Laboratory of Metal Matrix Composites, 800 Dongchuan Road, Shanghai 200240, China.

[§]Chulalongkorn University, Faculty of Engineering, Department of Chemical Engineering Research Unit in Polymeric Materials for Medical Practice Devices, 254 Phayathai Rd, Bangkok 10330, Thailand.

[£]University of Maribor, Faculty of Medicine, Department of Pharmacology, Taborska ulica 8, 2000 Maribor, Slovenia.

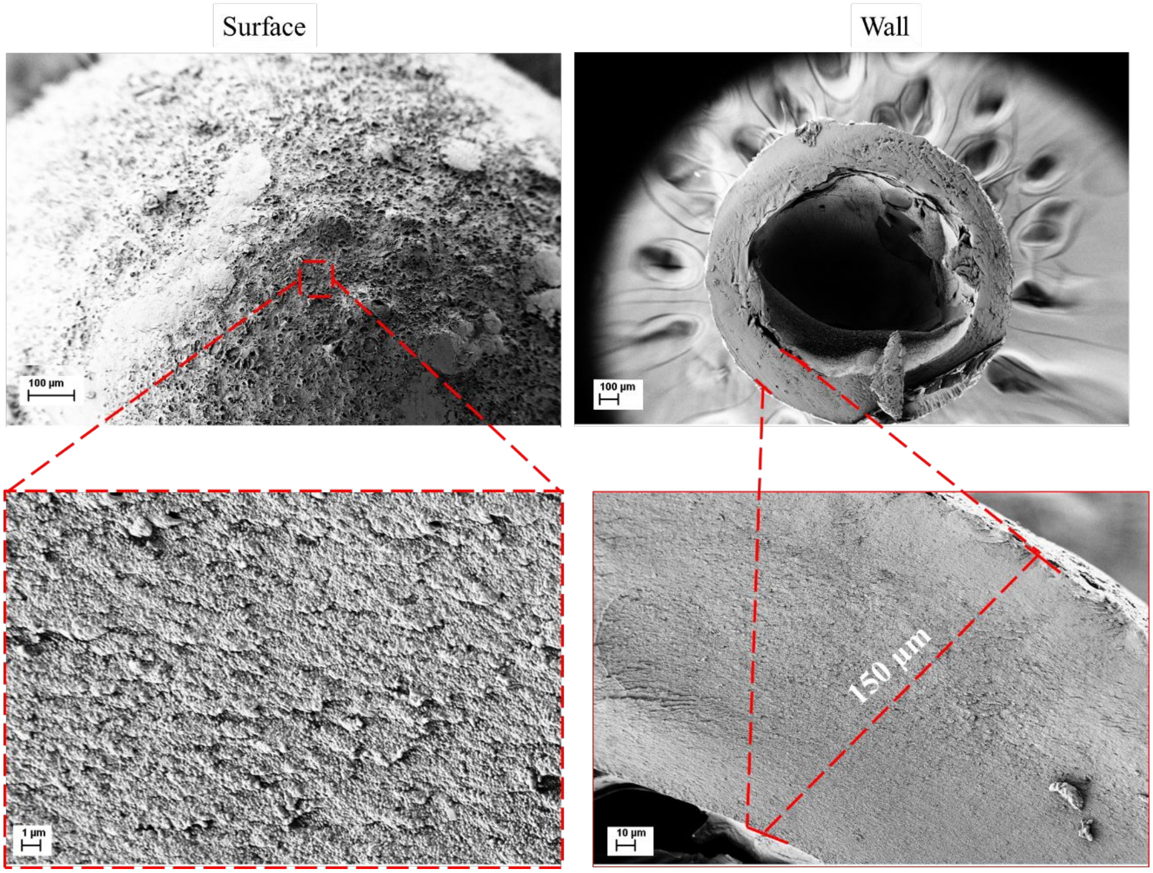

Figure S1. SEM images of cellulose acetate beads after treatment with 5 M potassium hydroxide solutions (KOH) at 90 °C.

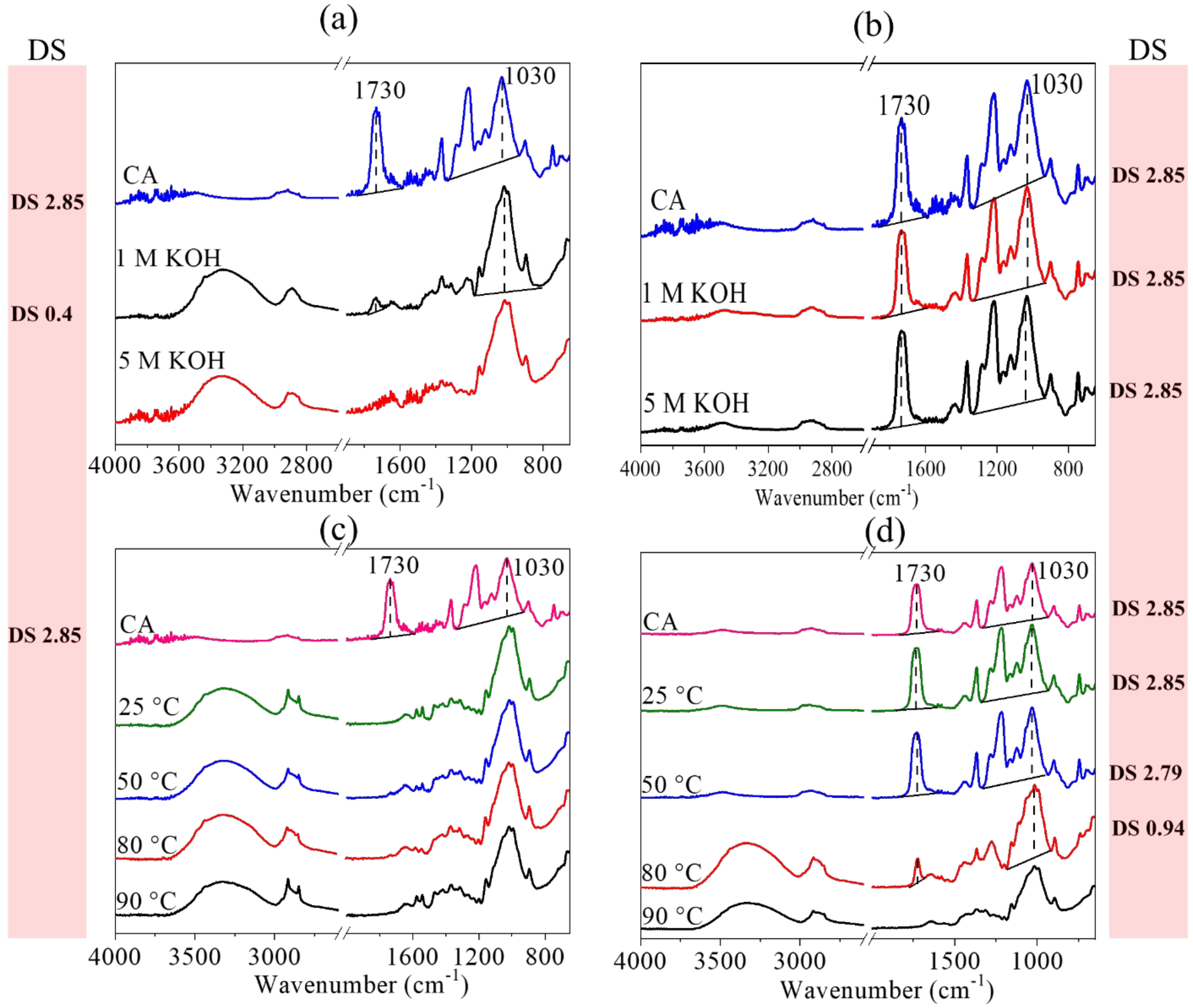

Figure S2. ATR-FTIR spectra and degree of substitution (DS) of cellulose acetate (CA) beads (a and c: Surface, b and d: Interior) before and after treatment with different concentrations of potassium hydroxide solutions (KOH) at an ambient temperature (25 °C, a and b), and with a 5 M KOH solution at different temperatures (25–90 °C, c and d) for 3 h.

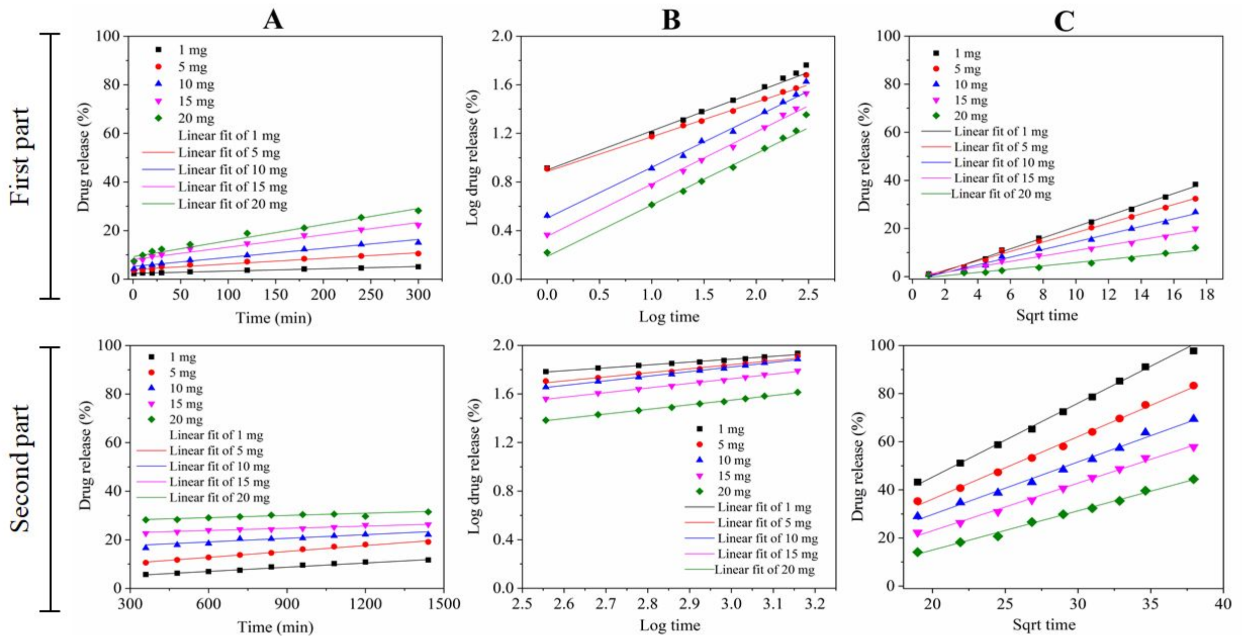

Figure S3. Drug release data of DCF-encapsulated DCF beads fitted to various kinetic models. (A) pH 1.2, zero-order kinetic, (B) pH 5.5, Higuchi-model and (C) pH 7.4, Korsmeyer-Peppas model.
